# Function of N6-Methyladenosine Modification in Tumors

**DOI:** 10.1155/2021/6461552

**Published:** 2021-11-23

**Authors:** Nan Zhang, Yuxin Zuo, Yu Peng, Lielian Zuo

**Affiliations:** Department of Physiology, Institute of Neuroscience Research, Hengyang Key Laboratory of Neurodegeneration and Cognitive Impairment, Hengyang Medical School, University of South China, 28 West Changsheng Road, Hengyang 421001, Hunan, China

## Abstract

N6-Methyladenosine (m6A) modification is a dynamic and reversible methylation modification at the N6-position of adenosine. As one of the most prevalent posttranscriptional methylation modifications of RNA, m6A modification participates in several mRNA processes, including nuclear export, splicing, translation, and degradation. Some proteins, such as METTL3, METTL14, WTAP, ALKBH5, FTO, and YTHDF1/2/3, are involved in methylation. These proteins are subdivided into writers (METTL3, METTL14, WTAP), erasers (ALKBH5, FTO), and readers (YTHDF1/2/3) according to their functions in m6A modification. Several studies have shown that abnormal m6A modification occurs in tumors, including colorectal cancer, liver cancer, breast cancer, nasopharyngeal carcinoma, and gastric cancer. The proteins for m6A modification are involved in tumor proliferation, angiogenesis, metastasis, immunity, and other processes. Herein, the roles of m6A modification in cancer are discussed, which will improve the understanding of tumorigenesis, as well as the diagnosis, treatment, and prognosis of tumors.

## 1. Introduction

Epigenetics is the study of gene expression regulated by modifications that do not alter the gene sequence. High-throughput sequencing showed that one-third to half of mRNA transcripts in humans and mice contain the m6A modification [1]. Recently, researchers have found that the m6A RNA modification participates as an epigenetic regulator that dynamically and reversibly controls the structure and function of RNA in tumors, including colorectal cancer, liver cancer, breast cancer, nasopharyngeal carcinoma, and gastric cancer [2–4]. The m6A modulates tumor proliferation, angiogenesis, metastasis, immunity, and other processes.

Writers, erasers, and readers regulate the methylation process involved in the m6A modification [3]. Writers and erasers transfer and remove the methylation groups, respectively. Readers recognize the adenosine bases that are modified by N6 methylation, thereby activating downstream regulatory pathways. This process involves multiple proteins. The m6A writer comprises a methyltransferase complex, which includes the core components of methyltransferase-like 3 (METTL3) and methyltransferase like 14 (METTL14), as well as WT1-associated protein (WTAP), KIAA1429, and other regulatory subunits [5, 6]. The main role of the writer is to catalyze the m6A modification of adenylate on mRNA. The erasers are demethylases, which reverse m6A modification and include *α*-ketoglutarate-dependent dioxygenase ALKB homolog 5 (ALKBH5) and fat-mass and obesity-associated protein (FTO) [7]. Readers recognize m6A modification sites and include YT521B homology domain-containing family protein 1/2/3 (YTHDF1/2/3), YTH domain-containing proteins 1/2 (YTHDC1/2), and members of the insulin-like growth factor 2 mRNA binding protein (IGF2BP) family [8].

In mammals, the m6A modification affects different aspects of RNA metabolism, including the regulation of mRNA stability, splicing, translation efficiency [1, 9, 10], nuclear export [11], selective polyadenylation [12], and T cell homeostasis [13]. The m6A modification was shown to participate in the regulation of gene expression, metabolism, and various other biological processes. Several studies have shown that m6A is involved in various regulatory mechanisms underlying cancer and may play an important role in carcinogenic initiation and progression [14].

In this review, we will discuss how the m6A modification participates in tumor proliferation, angiogenesis, invasion, and immunity and impacts cancer therapy. We will also discuss how this modification and its regulatory proteins can be used for targeted clinical treatment and provide suggestions for follow-up research.

## 2. M6A Modification and Proliferation

The m6A modification regulates the proliferation of various tumors, such as colorectal cancer, liver cancer, and breast cancer.

**Figure 1:**
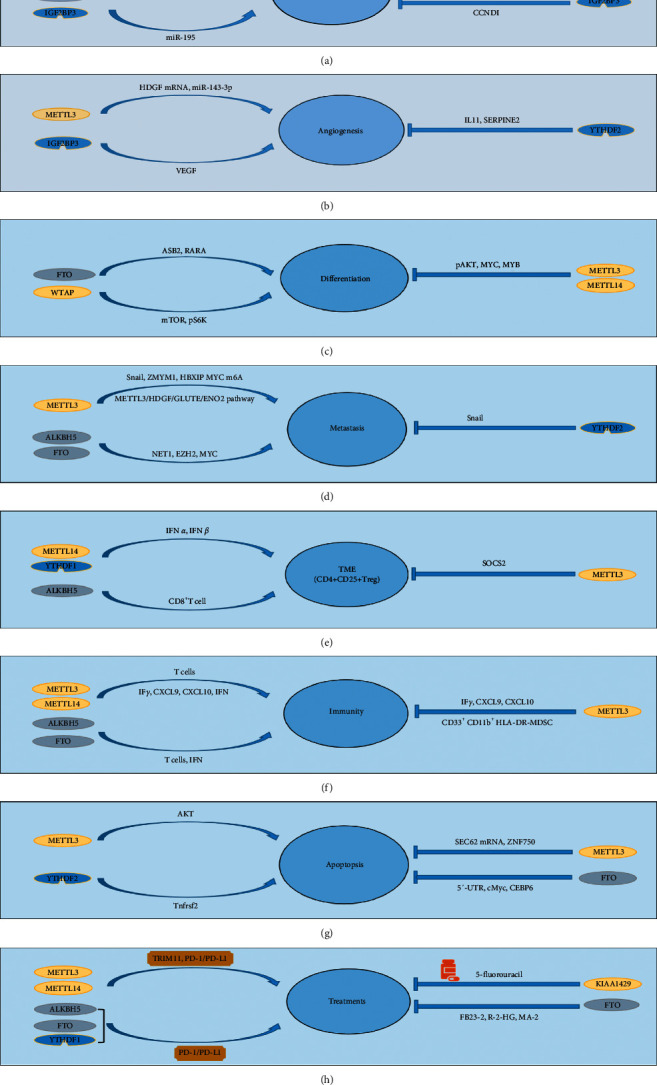
The function of the m6A RNA modification in cancer. (a–g) The m6A RNA modification can promote or inhibit cancer proliferation, angiogenesis, differentiation, metastasis, TME, immunity, and apoptosis through the “writers” METTL3, METTL14, WTAP, and KIAA1429,“ erasers” ALKBH5 and FTO, and “readers” YTHDF2 and IGF2BP3. (h) The m6A RNA modification can be involved in the clinical application of cancer, including cancer diagnosis and targeted treatments.

METTL3 was significantly elevated and promoted the proliferation of tumor cells by suppressing the expression of the suppressor of the cytokine signaling 2 gene in colorectal and liver cancers [15]. It has been recently revealed that m6A is also present in lncRNA. For instance, the lncRNA X-inactive specific transcript (XIST) is the downstream target of METTL14. The knockdown of METTL14 markedly augmented the proliferative and invasive abilities of colorectal cancer cells. METTL14 enhanced the m6A level in the lncRNA XIST and reduced its expression. It was also found that m6A-methylated XIST RNA was recognized by YTHDF2, which resulted in the degradation of XIST [16]. In addition, the concentration of m6A can also regulate mitosis, which affects cancer cell proliferation. By recognizing m6A modification in cyclin D1 mRNA, IGF2BP3 inhibited DNA replication in the S phase and the proliferation of colorectal cancer cells (Figure 1(a)) [17].

Notably, abnormal microRNA is involved in several disease development processes, such as tumor cell proliferation, metastasis, differentiation, and immunity [18, 19]. Studies have reported that m6A, an important posttranscriptional regulator in colorectal cancer (CRC) pathogenesis, is also associated with microRNA to promote CRC proliferation. Ye et al. observed that significantly increased IGF2BP2 increased the stability of RAF1 mRNA by blocking miR-195, which in turn promoted the proliferation of CRC cells [20]. MiR-96 directly targets AMPK*α*2, leading to increased expression of FTO and subsequent activation of MYC to promote the proliferation of CRC cells (Figure 1(a)) [21].

According to reports, the mature RNA stabilizer HuR regulates the stability of demethylated mRNA by blocking the binding of miRNA to 3ʹUTRs [22, 23]. HuR is considered an indirect m6A effector and is associated with m6A in liver cancer. The overexpression of WTAP in liver cancer contributes to the m6A modification of ETS1 mRNA, followed by apparent silencing of ETS1 in a HuR-related manner through the HuR-ETS1-p21/p27 axis, regulation of the G2/M phase, and increased progression [24]. KIAA1429 preferentially induces the m6A modification of the 3ʹUTR of GATA3 pre-mRNA in liver cancer cells, isolates HuR, degrades GATA3 pre-mRNA, downregulates the expression of GATA3, and, hence, promotes cancer growth [25]. However, Huang et al. found that IGF2BPs recruited HuR to stabilize RNA containing m6A modification [26]. Therefore, elucidation of the complicated regulatory mechanism involving HuR and m6A modifications requires further research (Figure 1(a)).

In breast cancer, the expressions of METTL3, METTL14, KIAA1429, and FTO are upregulated. METTL3 increases the expression of Bcl-2 mRNA [27] and downregulates the expression of the cell cycle inhibitor p21 [28]. FTO mediates the demethylation of tumor suppressor BNIP3 mRNA and induces its degradation via YTHDF2 [29]. Lnc942 directly binds to METTL14 by harboring a specific METTL14 binding domain (+176-+265), thereby stabilizing the expression and translation of downstream targets, such as CXCR4 and CYP1B1, and promoting the proliferation of tumor cells [30] (Figure 1(a)).

## 3. M6A Modification and Angiogenesis

Angiogenesis is critical for the development and progression of tumors. It is the key to rapid tumor proliferation. Vascular endothelial growth factor (VEGF) is at the frontline of this process. VEGF is an angiogenic factor secreted by tumor cells or lymphocytes and has been proven to be a primary factor in tumor angiogenesis.

In colon cancer cells, the m6A reader IGF2BP3 recognizes and binds to m6A modification sites in VEGF mRNA and promotes stability and expression. Reducing the expression of IGF2BP3 inhibits angiogenesis by downregulating VEGF [17]. In lung cancer tissues, METTL3 can trigger the splicing of the precursor miR-143-3p to generate mature miR-143-3p, which targets three binding sites in the vasohibin (VASH) 1 promoter and inhibits its expression. VASH1 was initially discovered to be the first of several endothelial cell factors that had an inhibitory effect on angiogenesis [31]. VASH1 mediates miR-143-3p-induced angiogenesis through the destabilization of the VEGFA protein. VASH1 can increase proteasomal degradation mediated by VEGFA ubiquitination in lung cancer cells [32]. However, the mechanism of ubiquitination of VEGFA induced by VASH1 has not been established and requires further studies. Hepatoma-derived growth factor (HDGF) mRNA is a key downstream target of METTL3 in gastric cancer (GC). METTL3 promotes m6A modifications of HDGF mRNA. IGF2BP3 directly recognizes and binds to the m6A sites and enhances the stability of HDGF. Increased secretion of HDGF promotes tumor angiogenesis [33] (Figure 1(b)).

In addition, when YTHDF2 is knocked out in liver cancer cells, it promotes the normalization of tumor blood vessels by increasing IL11 and SERPINE2 mRNAs. Hypoxia-inducible factor (HIF)-1*α* and HIF-2*α* play complementary roles in early angiogenesis, and vascular remodeling is mainly driven by HIF-2*α* [34]. In the case of hypoxia, HIF-2*α* is abnormally expressed, which leads to the downregulation of the expression of YTHDF2 and promotion of angiogenesis [35] (Figure 1(b)).

Cabozantinib exerts antitumor effects by targeting VEGFR-2 and inhibiting angiogenesis [36]. Regorafenib also inhibits tumor angiogenesis by acting on VEGF and has shown significant survival benefits as second-line therapy in treating hepatocellular carcinoma [37]. There are also classic drugs for solid tumor treatment, such as bevacizumab and ramucirumab, which work by acting on tumor angiogenesis [38, 39]. However, whether these drugs exert their antitumor angiogenic effects through some potential mechanisms related to m6A deserves deeper investigation in the future.

## 4. M6A Modifications and Differentiation

Recent studies have shown that m6A modification in mRNA or noncoding RNA plays an important role in the self-renewal and differentiation of stem cells [40]. Acute myelogenous leukemia (AML) is a type of malignant clonal disease of hematopoietic stem cells. Terminal differentiation arrest and abnormal proliferation of bone marrow cells are caused by bone marrow cells with the biological characteristics of AML [41]. In recent years, several studies have shown that m6A has a significant effect on the differentiation of AML. These include METTL3, METTL14, WTAP, and FTO. METTL3 promotes the growth of leukemia cells by inhibiting the differentiation of hematopoietic stem/progenitor cells, while knocking out METTL3 induces cell differentiation and delays the progression of leukemia in recipient mice [42]. Similarly, silencing METTL14 can induce the terminal differentiation of myeloid cells [41]. WTAP is overexpressed in 32% of AML patients. Studies have found that the knockout of WTAP inhibits the phosphorylation level of mTOR and its downstream target, p70 ribosomal subunit 6 kinase (pS6K), which is related to the promotion of myeloid differentiation of tumor cells [43]. FTO reduces the concentration of m6A in mRNA transcripts, regulates the expression of targets such as ASB2 and RARA, and inhibits the differentiation of AML cells induced by ATRA to promote the development of leukemia [40] (Figure 1(c)). m6A is also involved in regulating the differentiation of glioma cells. Visvanathan et al. found that the expression of METTL3 was weakened during the differentiation of GSCs, while Cui et al. found that m6A concentrations significantly increased during the induced differentiation of GSCs. This difference may be due to different pathways acting on different molecular mechanism pathways, leading to differential expression results, which need to be further explored [44, 45].

## 5. M6A Modification and Metastasis

Metastasis is related to a poor prognosis and high mortality in patients with cancer. The role of m6A modifications in cancer metastasis has been a hot research topic. Epithelial-mesenchymal transition (EMT) is an important step in cancer metastasis. It is a complex process that includes not only the reduction of epithelial cell-cell interactions, but also the loss of epithelial cell polarity [46]. The expression of E-cadherin is considered a marker of EMT [47]. Studies have shown that the transcription factor Snail is a key transcriptional inhibitor of E-cadherin expression during EMT [48]. The m6A regulated the metastasis of cancers. METTL3 is upregulated in most tumors, including nasopharyngeal [49], gastric [50], liver [51], and bladder cancers [52], but it is downregulated in colorectal cancer [53]. The differential expression of METTL3 among tumors plays a dual role, which may reflect differences in targeting pathways and tumor heterogeneity. Xinyao et al. found that knocking out METTL3 downregulated the expression of Snail, thereby reducing the in vitro invasiveness and EMT concentrations in liver cancer cells [49]. The overexpression of YTHDF2 reduced the expression and stability of mature Snail mRNA in HeLa cells [54] (Figure 1(d)). The metastatic mechanisms of METTL3 and YTHDF2 need to be investigated in the future.

In GC, METTL3 modified ZMYM1 mRNA with m6A and enhanced its stability and expression. ZMYM1 then recruited the CTBP/LSD1/COREST complex to bind to the E-cadherin promoter and inhibit its expression, which promoted EMT and GC metastasis [50]. As the most common malignant tumor of the digestive system, GC is highly metastatic. Acetylation of H3K27 is mediated by p300 activation in the METTL3 promoter, which induces the transcription of METTL3 and promotes the occurrence of GC and liver metastasis through the METTL3/HDGF/GLUT4/ENO2 pathway [33]. ALKBH5 reduced NEAT1 m6A modifications through demethylation, resulting in increased NEAT1 concentrations and the promotion of EZH2 overexpression [55]. FTO promoted the expression of MYC by reducing the m6A modification of MYC and ultimately leading to enhanced metastasis and invasion of GC cells [56]. In addition, silencing HBXIP reduced the expression of METTL3 and reduced MYC m6A mRNA modification and translation, thereby inhibiting the metastasis and invasion of GC cells [57] (Figure 1(d)). This study could not verify whether HBXIP acted as a transcription factor of METTL3, which should be addressed in further studies.

## 6. M6A Modification and Tumor Microenvironment

The tumor microenvironment (TME) is a dynamic and complex system with several components, including fibroblasts, immune cells, and lymphocytes, that play a key role in the development and progression of cancer. The interaction between tumor cells and the TME is key for the immune escape of tumor cells through the depletion of antigen-presenting cells (APCs), high-level recruited or induced inhibitory immune cells, such as CD4^+^ regulatory T cells (Tregs), bone marrow-derived suppressor cells (MDSCs), and various cytokines [58–60]. It has been shown that m6A RNA modifications occurring in the TME are important for mediating tumor progression and influencing tumor treatment outcomes [61, 62].

Recently, CD4^+^CD25^+^ Foxp3 Treg has been highlighted as playing a key immunosuppressive role in the cellular subpopulation of the tumor microenvironment in hepatocellular carcinoma (HCC), which is critical in both the development and spread of HCC [63]. Overexpression of METTL3 suppresses the expression of cytokine signaling factor 2 (SOCS2) through an m6A-YTHDF2-dependent mechanism, which in turn regulates the immunosuppression of CD4^+^CD25^+^ Foxp3Treg cells involved in TME and ultimately promotes hepatocellular carcinoma cells growth (Figure 1(e)) [64, 65].

The association of m6A with the tumor microenvironment was also observed in pancreatic cancer. The deletion of ALKBH5 decreased the infiltration of CD8^+^ T cells in pancreatic cancers [66]. In the established m6A-related lncRNA prognostic risk score-based high-risk group of patients with pancreatic ductal adenocarcinoma (PDAC), significantly elevated levels of plasma B cell and resting NK-cell infiltration were observed, while resting memory CD4 T cells, monocytes, and resting mast cell infiltration levels were significantly decreased (Figure 1(e)) [67].

Transcript levels of IFN-*α*, IFN-*β*, and IFN-*γ* were observed to be downregulated after knockdown of METTL14/YTHDF1 in gastric cancer cell lines, while IFN-*α* and IFN-*β* were involved in promoting the accumulation of pDCs in TME and the expression of PD-L1 in CD4^+^CD25^+^Tregs [68–70]. In addition, Zhang et al. identified three different m6A modification patterns, which characterized TME cell infiltration in three patterns that were highly consistent with three immunophenotypes of tumor immune rejection, immune inflammation, and immune desert. This also demonstrates the importance of m6A in shaping the immune microenvironment of different tumors (Figure 1(e)) [71].

## 7. M6A Modification and Immunity

m6A RNA modification plays an important role in tumor immunity. METTL3 promotes the translation of CD40, CD80, and the TLR4 signal adaptor TIRAP and enhances the activation of dendritic cells (DCs) and T cells in vitro [72]. In mouse T cells, the deletion of METTL3 inhibited the IL-7/STAT5/SOCS pathway, which reduced homeostasis and differentiation [13]. Furthermore, the reduction of METTL3 expression in DCs can lead to DC maturation disorders, which, in turn, decreases the levels of the costimulatory molecules CD40, CD80, and interleukin-12 and weakens the activation of T cells in vitro [72] (Figure 1(f)).

METTL3 can directly induce CD33^+^CD11b^+^HLA-DR-myeloid-derived suppressor cell (MDSC) differentiation or cervical squamous cell carcinoma-related MDSC differentiation in vitro. The tumor-infiltrating MDSC population inhibits the proliferation and function of T cells and induces immune tolerance for cervical squamous cell carcinoma [73]. In colorectal cancer, the lack of METTL3 or METTL14 increases cytotoxic tumor-infiltrating CD8+ T cells; enhances the production of interferon- (IFN-) *γ*, CXCL9, and CXCL10; and promotes the recruitment of CD8+ and CD4+ effector T cells that inhibit tumor growth [74] (Figure 1(f)).

IFN is a glycoprotein with immunomodulatory, antitumor, antiviral, and cell proliferation activities. In studies involving m6A, it was found that the production of type I IFN triggered by dsDNA or human cytomegalovirus (HCMV) was controlled by the m6A methyltransferase subunit METTL14 and demethylase ALKBH5. Silencing METTL14 with siRNA reduced HCMV reproduction and stimulated the accumulation of IFNB1 mRNA after exposure to dsDNA or HCMV, while silencing ALKBH5 showed the opposite effect [75] (Figure 1(a)). The replication of human immunodeficiency and influenza viruses is also affected by the METTL3/14 m6A writer complex, but the specific mechanism needs to be further studied (Figure 1(f)) [76].

In melanoma cells, IFN-*γ* downregulates FTO, and FTO promotes resistance to IFN-*γ*-mediated killing through its m6A demethylase activity and downstream targets PD-1 (PDCD-1), CXCR4, and SOX10 [77]. In liver cancer, hepatitis B virus (HBV) promotes the m6A modification of host PTEN mRNA, resulting in reduced RNA stability and decreased expression. The PI3K/AKT pathway can be activated by HBV and affect the synthesis of IFN, inhibit innate immunity, and promote the development of liver cancer [78]. Interestingly, IFN-*α* 2a can increase the m6A RNA modification level of HBV pgRNA and reduce its stability, thereby inhibiting the development of HBV-related liver cancer (Figure 1(f)) [79].

## 8. M6A Modifications and Apoptosis

Apoptosis is a common biological phenomenon of cells and plays an important role in the development of several systems. m6A is involved in the development of tumors and regulates apoptosis of tumors.

The miR-4429 targeted and inhibited METTL3 to reduce the level of m6A in SEC62 mRNA. Finally, this result reduced the stabilizing effect of IGF2BP1 on SEC62 mRNA to facilitate apoptosis of GC cells [80]. Further research showed that silencing HBXIP leads to a decrease in the expression of METTL3 and promotes apoptosis of GC cells [57]. The consumption of METTL3 leads to an increase in the level of phosphorylated AKT, induces apoptosis, and delays the progression of mouse leukemia [42]. Similarly, METTL3 inhibits the expression of ZNF750 and then promotes apoptosis through the NF750-FGF14 signal axis in nasopharyngeal carcinoma [81]. In lung cancer cells, knocking out METTL3 can increase the apoptosis of tumor cells to suppress the tumorigenicity of cancer cells [82] (Figure 1(g)). However, the mechanisms involved need to be further investigated.

R-2-Hydroxyglutarate (R-2HG) directly binds to the FTO (which participates in the m6A process) and inhibits its activity, thereby reducing the m6A modification and stability of c-MYC and CEBPA in the 5′-UTR and coding regions, leading to apoptosis of leukemia cells [83]. Another study showed that FB23 can suppress FTO and significantly promote the apoptosis of AML cells in vitro [84]. YTHDF2 protein reduces the half-life of various m6A transcripts, which contribute to the integrity of leukemic stem cell (LSC) function, including the tumor necrosis factor receptor, Tnfrsf2, which is upregulated in YTHDF2-deficient LSCs to initiate apoptosis [85] (Figure 1(g)).

## 9. M6A Modifications and Clinical Applications

In recent years, with the in-depth research on m6A RNA modifications in the field of cancer, certain progress has been made in drug therapy, and there are related clinical applications. IGF2BP3 may be used as a prognostic indicator of colon cancer [17]. KIAA1429 [25] and WTAP [24] may be prognostic indicators for patients with HCC.

FTO is an important marker of leukemia. In in vitro experiments, the FTO inhibitor FB23-2 reduced the expression of FTO and significantly inhibited the proliferation of human acute myeloid leukemia primary cells [84]. R-2-Hydroxyglutarate (R-2-HG) can also inhibit FTO activity, thereby increasing the m6A RNA modification in R-2HG-sensitive leukemia cells, reducing the stability of MYC/CEBPA transcripts, and leading to the inhibition of related pathways. R-2HG also inhibits the proliferation and vitality of leukemia cells by promoting cell cycle arrest and exerts several antileukemia activities in vivo and in vitro [83]. In vivo treatment of glioma with the FTO inhibitor MA-2 (an ethyl ester meclofenamic acid derivative) has been shown to inhibit the growth of glioblastoma stem cells (GSCs) and GSC-induced tumorigenesis [45]. METTL3 enhances DNA repair through a SOX2-dependent pathway and reduces the sensitivity of GSCs to gamma radiation [44] (Figure 1(h)).

Both metformin and 5′-fluorouracil are important therapeutic drugs for treating breast cancer. Metformin, a well-known drug repurposing candidate for breast cancer, exhibits antiproliferative activity in breast cancer cells through the miR-483-3p/METTL3/m6A/p21 pathway [28]. The drug 5′-fluorouracil significantly reduces the expression of KIAA1429 and CDK1 mRNA and protein levels, thereby reducing proliferation and metastasis of breast cancer [86] (Figure 1(h)).

PD-1 and PD-L1, which are important proteins in tumor immunity, have become vital targets for tumor immunotherapy [87]. The m6A scoring system developed by Du et al. is significantly related to the response to anti-PD-1/PD-L1 immunotherapy. This scoring system aids in the clinical identification of patients who may be sensitive to PD-1/PD-L1 immune checkpoint blockade and those patients who are suitable for immunotherapy, resulting in higher therapeutic benefits [88]. Inhibiting the interaction between PD-1 and PD-L1 can enhance T cell responses in vitro and mediate preclinical antitumor activity. This is called the immune checkpoint blockade treatment effect. Silencing YTHDF1 can enhance the PD-L1 checkpoint blockade [89]. ALKBH5 enhances anti-PD-1 immunotherapy by regulating the content of lactic acid and accumulation of tumor immune cells in the TME [90]. The loss of METTL3 or METTL14 was shown to enhance the sensitivity of colorectal cancer and melanoma to anti-PD-1 treatment [74]. Additionally, knockout of FTO sensitizes mouse melanomas to anti-PD-1 treatment [77] (Figure 1(h)).

The development of drug resistance is common during cancer treatment. In drug-resistant nasopharyngeal carcinoma cells, the m6A modification of TRIM11 mediated by METTL3 promotes the stability of TRIM11 mRNA through the m6A-IGF2BP2-dependent pathway, which partially increases the expression of the TRIM11 protein. Upregulation of TRIM11 can further activate the *β*-catenin/ABCC9 axis, which promotes resistance to chemotherapy in nasopharyngeal carcinoma [91]. Downregulation of FTO and ALKBH5 can increase the activation of the Wnt/*β*-catenin signaling pathway by m6A modification of FZD10 mRNA and promote the resistance of BRCA-deficient epithelial ovarian cancer cells to poly (ADP-ribose) polymerase inhibitors [92] (Figure 1(h)).

RNA methylation analysis is valuable for cancer prognosis. Qiang et al. found that the expression of METTL3 is a potential prognostic marker for human GC [33]. Furthermore, Jingnan et al. found that the reader YTHDF1 may also be useful as a prognostic marker for GC [8]. Kelei et al. determined that GC with the high expression of WTAP and FTO indicated a poor prognosis for these patients. Cox regression analysis indicated that the m6A risk score is a prognostic factor for overall survival, and the upregulation of FTO may be a potential independent prognostic factor for recurrence-free survival in patients with GC [93].

## 10. Conclusions

As the most common mRNA modification, m6A RNA modification is a complex cellular process that regulates the development of diseases. With the emergence of highly specific antibodies and the popularization of high-throughput sequencing technologies, studies on the role of m6A in the development of cancer have increased rapidly. So far, only a few mechanisms underlying the role of m6A are understood. However, there are more mechanisms of m6A that need to be further investigated to facilitate the discovery of potential targets for the diagnosis and treatment of tumors.

## Data Availability

No raw data were associated in this review.

## Abbreviations

m6A: N6-Methyladenosine
METTL3: Methyltransferase-like 3
METTLE4: Methyltransferase-like 4
WTAP: WT1-associated protein
ALKBH5: ALKB homolog 5
FTO: Fat-mass and obesity-associated protein
YTHDF1/2/3: YT521B homology domain-containing family protein 1/2/3
YTHDC1/2: YTH domain-containing proteins 1/2
IGF2BP: Insulin-like growth factor 2 mRNA binding protein
XIST: X-inactive specific transcript
HIF: Hypoxia-inducible factor
VEGF: Vascular endothelial growth factor
HDGF: Hepatoma-derived growth factor
GC: Gastric cancer
EMT: Epithelial-mesenchymal transition
DCs: Dendritic cells
MDSC: Myeloid-derived suppressor cell
HCMV: Human cytomegalovirus
HBV: Hepatitis B virus
R-2-HG: R-2-Hydroxyglutarate
GSCs: Glioblastoma stem cells
pS6K: p70 ribosomal subunit 6 kinase
TME: Tumor microenvironment
APC: Antigen-presenting cells
SOCS2: Suppressor of cytokine signaling factor 2
PDAC: Pancreatic ductal adenocarcinoma
CRC: Colorectal cancer.
