# Corrigendum to “Function of N6-Methyladenosine Modification in Tumors”

**DOI:** 10.1155/2023/9870174

**Published:** 2023-10-10

**Authors:** Nan Zhang, Yuxin Zuo, Yu Peng, Lielian Zuo

**Affiliations:** Department of Physiology, Institute of Neuroscience Research, Hengyang Key Laboratory of Neurodegeneration and Cognitive Impairment, Hengyang Medical School, University of South China, 28 West Changsheng Road, Hengyang 421001, Hunan, China

In the article titled “Function of N6-Methyladenosine Modification in Tumors” [1], there was an error in the Acknowledgments section. This is corrected as shown below.

This work was supported by grants from the National Natural Science Foundation of China (no. 81903030), Outstanding Young Aid Program for Education Department of Hunan Province (Grant no. 20B511), the Natural Science Foundation of Hunan Province, China (no. 2021JJ40481), and Graduate Student Scientific Research Innovation Project of University of South China (no. S202110555298).
